# Semi-Automated Lung Segmentation Based on Region-Growing Methods in Interstitial Lung Disease

**DOI:** 10.3390/jcm15041339

**Published:** 2026-02-08

**Authors:** Mădălin-Cristian Moraru, Cristiana-Iulia Dumitrescu, Suzana Măceș, Cătălin Ciobîrcă, Mihai Popescu, Luana Corina Lascu, Dragoș-Ovidiu Alexandru, Diana-Maria Trască, Diana Maria Ciobîrcă, Marian-Răzvan Bălan, Oana Sorina Tica, Radu Teodoru Popa, Daniela Dumitrescu

**Affiliations:** 1 Doctoral School, University of Medicine and Pharmacy of Craiova, 200349 Craiova, Romania; madalin.moraru@umfcv.ro (M.-C.M.); balan.razvan796@gmail.com (M.-R.B.); 2 SC SPAD IMAGING INTERNATIONAL Center Craiova, 200352 Craiova, Romania; catalin.ciobirca@yahoo.com (C.C.); mihai.popescu@umfcv.ro (M.P.); luana.lascu@umfcv.ro (L.C.L.); daniela.dumitrescu@umfcv.ro (D.D.); 3 Department of Pharmacology, Faculty of Medicine, University of Medicine and Pharmacy of Craiova, 200349 Craiova, Romania; 4 Department of Radiology and Medical Imaging, University of Medicine and Pharmacy of Craiova, 200349 Craiova, Romania; 5 Department of Radiology and Medical Imaging, University Emergency County Clinical Hospital, 200642 Craiova, Romania; dragos.alexandru@umfcv.ro; 6 Department of Medical Informatics and Biostatistics, Faculty of Medicine, University of Medicine and Pharmacy of Craiova, 200349 Craiova, Romania; 7 Department of Internal Medicine, University of Medicine and Pharmacy of Craiova, 200349 Craiova, Romania; diana.trasca@umfcv.ro; 8 Slatina Emergency County Hospital, 230008 Slatina, Romania; diana.ciobirca@yahoo.com; 9 Department of Radiotherapy, University Emergency County Clinical Hospital Craiova, 200642 Craiova, Romania; 10 Department of Mother and Child, University of Medicine and Pharmacy of Craiova, 200349 Craiova, Romania; oanabanica25@yahoo.com; 11 Department of Computer Science, University of Craiova, 200585 Craiova, Romania; popateodoru@yahoo.com

**Keywords:** lung segmentation, region growing, semi-automated segmentation, quantitative imaging, high-resolution CT, histogram analysis, planning software, pulmonary fibrosis, interstitial lung disease

## Abstract

**Background**: One of the main tools for investigating pulmonary disorders is computed tomography. Starting with a CT, analyses can be qualitative (e.g., direct interpretation of 2D slices, virtual bronchoscopy) or quantitative (e.g., fibrosis score). Qualitative analyses can be performed without segmentation, but quantitative analyses require lung segmentation. **Methods**: We present the concepts for a class of lung segmentation methods that use region-growing algorithms, the implementation and testing details, and the results obtained in our software platform. Accurate segmentation of lung regions from medical images is a crucial step in computer-aided diagnosis (CAD) systems for pulmonary diseases such as chronic obstructive pulmonary disease (COPD), pneumonia, and lung cancer. Manual segmentation is time-consuming and subjective, while fully automated methods may fail under challenging imaging conditions. **Results**: This article presents a semi-automated lung segmentation approach, based on region-growing methods, that balances automation with user control. **Conclusions**: The proposed technique effectively delineates lung boundaries in computed tomography (CT), minimizing computational complexity and manual effort.

## 1. Introduction

Interstitial lung diseases (ILDs) represent a heterogeneous group of parenchymal lung disorders characterized by inflammatory and fibrotic remodeling of the pulmonary interstitium, progressive architectural distortion, and decline in respiratory function. The irreversible accumulation of fibrotic tissue results in the loss of lung elasticity and reduced lung compliance, as well as compromised gas exchange, ultimately presenting with symptoms of dyspnea on exertion, chronic cough, and reduction in exercise tolerance. The etiology of ILDs includes, but is not limited to, connective tissue disease, environmental/occupational exposure, and drug-induced ILD, as well as idiopathic forms such as idiopathic pulmonary fibrosis (IPF), which carries a poor prognosis and a highly variable disease course among affected individuals [1,2,3,4].

High-resolution computed tomography (HRCT) is the gold standard for ILD assessment, enabling identification of specific morphological patterns—such as reticular opacities, ground-glass attenuation, subpleural fibrosis, traction bronchiectasis, architectural distortion, and honeycombing—that are essential for disease categorization, risk stratification, management, and follow-up [5]. Beyond qualitative radiologic evaluation, HRCT also enables quantitative imaging, providing objective, reproducible measurements of lung pathology for clinical and research applications.

Conventional segmentation methods, including global thresholding, edge detection, region-growing, and clustering, are often robust in the case of a healthy lung but are likely to fail in ILD cases due to heterogeneity of lung structure, richness of the vascular tree, artifacts, or a shift in intensity caused by the presence of fibrosis, traction bronchiectasis, and contact with the pleura that may result in under-segmentation or leaking into the mediastinum or chest wall, or failure to include diseased areas, which may invalidate quantitative features [6,7,8]. Fully automated or deep learning-based methods have been proposed, but they require a large number of labeled images, may yield unpredictable results for challenging pathologies, and do not allow experts to correct or fine-tune results when needed [9,10,11].

Consequently, semi-automated segmentation techniques remain of great interest for scientific studies and clinical evaluation, as they balance automation with manual control. Among semi-automated segmentation techniques, region-growing is among the most appropriate for lung segmentation, as it is intuitive, can handle spatial continuity, and performs well in the relatively homogeneous regions of lung parenchyma. It is a neighborhood-dependent technique that iteratively incorporates neighboring pixels or voxels into the segmented area, enabling adaptation of the segmentation to the local intensity distribution while preserving anatomical continuity [12,13]. However, most existing algorithms impose a limit on the intensity range and may, therefore, miss vessels or disease processes confined within the lung, which could reduce the capabilities of subsequent analysis [14].

To address these limitations, the present study proposes a semi-automated lung segmentation approach based on a 3D region-growing algorithm incorporating an embeddedness-based selection criterion designed to preserve the complete internal intensity spectrum of the lung volume—including air, normal parenchyma, vascular structures, and fibrotic tissue—while truncating protruding extensions outside the lung boundaries. This property enables reliable post-segmentation histogram evaluation and supports the development of quantitative fibrosis-related image biomarkers. Here, we outline the method’s core principles, practical implementation, and qualitative validation using simulated and anonymized ILD CT images. The goal of this study is to enhance the robustness, interpretability, and clinical relevance of quantitative image analysis techniques.

## 2. Materials and Methods

### 2.1. Region-Growing Technique Overview

3D region-growing is a voxel-based image segmentation approach that groups neighboring voxels with similar intensity and/or geometric properties (gradient, second-order differences, feature closeness, etc.) to form regions [15].

The process typically includes the following steps:

1. Seed Selection: The user selects one or more seed points within the region of interest (e.g., lung parenchyma).
2. Growth Criteria: A homogeneity criterion (e.g., intensity difference threshold) determines whether adjacent voxels belong to the same region.
3. Region Expansion: At each iteration, the algorithm includes neighboring voxels that meet the similarity condition until no more voxels satisfy the growth criteria.
4. Post-Processing: Morphological operations (e.g., hole filling, smoothing, and edge refinement) are applied to improve segmentation quality.

The main advantage of region-growing is its intuitive nature and high accuracy in homogeneous regions, making it well-suited for lung segmentation where the tissue intensity is relatively uniform compared to surrounding structures [8,14].

### 2.2. Methodology

Lung fibrosis appears on CT scans with several characteristic features:

- Ground-glass opacities are areas of increased opacity that can indicate inflammation or early fibrosis. They appear hazy on the CT scan but still allow the underlying structures to be seen [16].
- It can have a reticular pattern that consists of a network of thin, irregular lines representing thickened interlobular septa and peribronchial areas. It is often seen in more established fibrosis [16,17].
- Honeycombing is a classic sign of advanced fibrosis, characterized by clustered cystic spaces with thickened walls, often at the periphery of the lungs. It resembles the appearance of a honeycomb and is a sign of significant scarring and lung damage [16].
- Traction bronchiectasis refers to the dilation of the bronchi (airways) due to the pulling force of the fibrotic tissue. It is often seen in combination with honeycombing and reticular patterns [16].
- Architectural distortion describes the distortion of the normal lung structure due to fibrotic changes. It can be associated with the aforementioned features and reflects a more advanced disease [16,18].

All these patterns and signs permit radiologists and clinicians to assess the extent and progression of lung fibrosis.

Our proposed semi-automated region-growing segmentation follows these steps:

1. Preprocessing:
  - Apply noise reduction filters such as Gaussian or median filtering.
  - Normalize image intensities to enhance contrast.
2. Seed-Point Selection:
  - The user manually selects a seed point inside the trachea.
  - Automatic seeding can be integrated using heuristic rules (e.g., selecting low-intensity regions within the thoracic cavity).
3. Region-Growing:
  - A similarity threshold T for the lung parenchyma and the air regions is established.
  - We define an “embeddedness” filter that detects if a voxel of any intensity is embedded into lung intensities (parenchyma or air).
  - The algorithm performs a BFS (breadth-first search) starting from the seed points and expands the region until boundary conditions are met and the “embeddedness” filter can no longer select voxels.
4. Morphological Refinement:
  - Apply morphological closing to remove small holes.
  - Optionally, exclude airways or trachea using shape filters.
5. Histogram computation and visualization.

As mentioned, in our implementation, the seed is only one point inside the trachea, manually selected by the user (note: this is the only manual operation of the method, performed in only a few seconds on the 2D CT sections). The region expansion is performed using a breadth-first search (BFS) strategy on a 6-connected voxel grid. Each voxel is visited at most once during propagation, ensuring stable growth and preventing uncontrolled leakage through narrow anatomical connections. This connectivity choice represents a balance between computational efficiency and anatomical continuity in volumetric CT data.

To ensure preservation of all anatomically relevant structures within the lung volume—irrespective of their attenuation values—we introduce an embeddedness-based voxel selection criterion. Unlike classical region-growing approaches that rely primarily on intensity thresholds, the proposed criterion evaluates the local spatial context of a voxel relative to surrounding lung tissue.

Let p denote a candidate voxel with arbitrary intensity. The voxel p is considered embedded within the lung volume if, along a sufficient number of axial directions, lung-range attenuation values are encountered within a bounded spatial neighborhood. For each of the six principal directions {±x, ±y, ±z}, a ray is cast from p and sampled up to a maximum probing distance R. If at least one voxel along the ray exhibits an intensity within a predefined lung witness interval [I_min, I_max], that direction is considered satisfied.

The voxel p is accepted if the number of unsatisfied directions does not exceed a maximum allowable miss count M. Formally, p is embedded if the number of satisfied directions is greater than or equal to |D| − M, where D denotes the set of axial directions. This criterion allows inclusion of voxels of any intensity provided they are structurally enclosed within lung tissue, thereby preventing leakage into surrounding mediastinal or chest wall regions while preserving internal pathological structures.

The embeddedness criterion is governed by a small set of intuitive parameters: (i) I_min and I_max, defining the lung witness intensity interval; (ii) R, the maximum probing distance along each direction; and (iii) M, the maximum number of allowed unsatisfied directions. In the experiments presented in this study, the following parameter values were used: I_min = −1500 HU, I_max = −200 HU, R = 5 voxels, M = 2, and a 6-connected neighborhood was employed for region expansion.

## 3. Results

Quantitative evaluation of segmentation performance was performed using overlap-based metrics, specifically the Dice similarity coefficient, computed against reference segmentations generated using established tools (3D Slicer 5.10.0, Lung CT Analyzer extension revision e2f23da, and TotalSegmentator 2.12.0). While manual expert annotations are often considered a reference standard, their reliability in advanced interstitial lung disease is limited due to high inter- and intra-observer variability. For this reason, automated and semi-automated reference segmentations were used to provide a consistent and reproducible baseline for quantitative comparison. In the context of interstitial lung disease, particularly in advanced fibrotic cases, manual delineation of the lung surface by human experts is inherently imprecise and subject to high inter- and intra-observer variability. Fibrotic remodeling, subpleural reticulation, traction bronchiectasis, and architectural distortion obscure clear anatomical boundaries, making it unrealistic to define a reliable voxel-wise ground-truth suitable for Dice-based evaluation. As a result, Dice scores computed against manual segmentations in such cases may reflect annotation uncertainty rather than true algorithmic performance.

**Figure 1:**
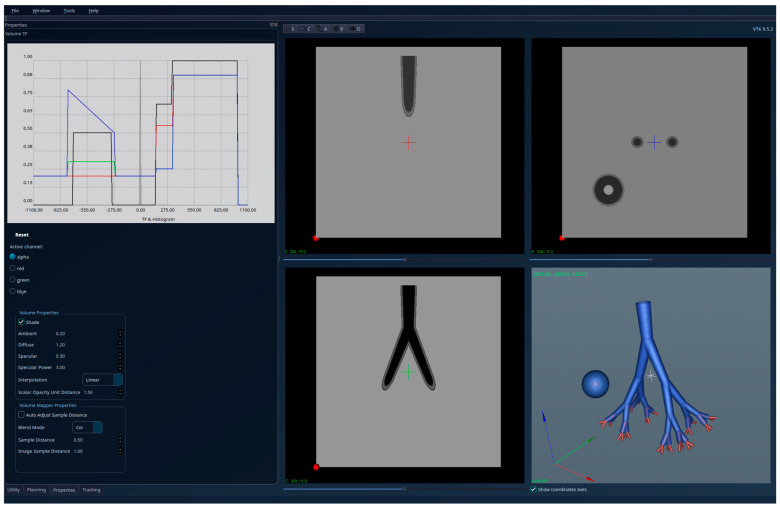
Theoretical lungs model—unsegmented (two separated regions with similar HU intensities, with one of them being a tracheobronchial tree with “red” structures-HU outside the lung parenchyma interval-protruding outside the lung).

**Figure 2:**
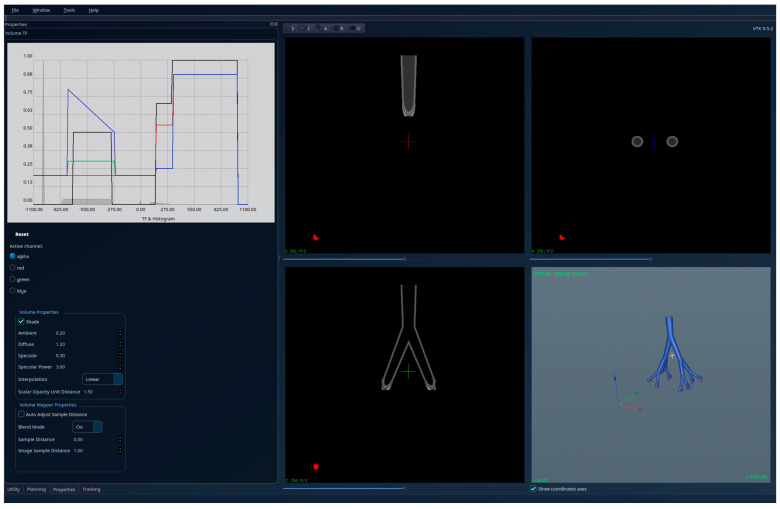
Theoretical lungs model-segmented; only the tracheobronchial is kept, and the protruding red regions are cut to the interior of the lungs’ volume.

To partially compensate for this limitation, accuracy was evaluated on a theoretical lung dataset with known ground-truth geometry (see Figure 1 and Figure 2). In this controlled setting, the proposed algorithm correctly retained only the connected regions that satisfied the embeddedness criterion, while excluding all protruding structures outside the lung volume, achieving 100% accuracy relative to the theoretical reference. This validation confirms the correctness of the embeddedness-based selection mechanism under idealized conditions, while a qualitative evaluation of real patient data demonstrates its robustness in clinically realistic, highly complex scenarios.

**Figure 3:**
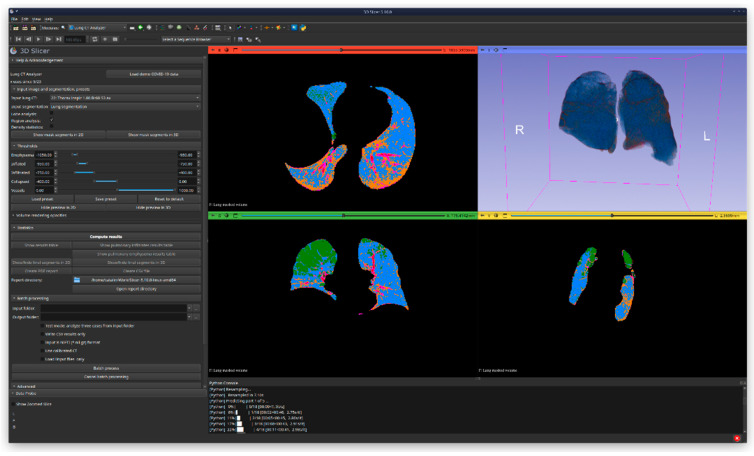
Patient 4 (emphysema)-Slicer segmentation results based on LungCTAnalyzer extension and TotalSegmentator_lung extended method.

One other evaluation was based on the Dice score computed against segmentations created by 3D Slicer [19], with the Lung CT Analyzer extension [20], and Total Segmentator [21]. The results of this evaluation are presented in Figure 3 (for one patient only) and in Table 1 (for all datasets). All the scores for the real datasets were greater than 0.95, while for the theoretical model, we could not compute a segmentation using Slicer 5.10.0.

Our method has the advantage of detecting the entire lung volume, including all its intensities: air regions, lung parenchyma, pathology (e.g., fibrosis, but not limited to), and blood vessels. Any characteristics that extend outside the lungs (e.g., blood vessels) are cut after the segmentation to the portions “embedded” into the lungs’ volume.

From a computational perspective, the algorithm operates in linear time with respect to the number of voxels in the input volume, as each voxel is processed at most once during region expansion, while the embeddedness evaluation incurs a constant number of local samples per voxel.

This post-segmentation conservation of all the intensities that are found inside the lungs’ volume on the input 3D image allows a pertinent analysis of the post-segmentation 3D image histogram. In the tests we performed, there are clear qualitative and quantitative differences between the healthy and fibrotic lungs’ histograms, and these observations can lead to future development in the computation of a pertinent fibrosis score.

The proposed method was implemented in the software platform presented in [22], which is based on the C++ programming language, the VTK, and Qt libraries. The general characteristics of the datasets used for the tests are shown in Table 2. For the initial validation of the algorithm’s implementation, we prepared a theoretical dataset similar to that reported in [23]—the difference was the presence of connected regions with high (HU) intensities that extend beyond the theoretical lung volume. For real medical validation, we used four anonymized datasets—one from a patient with healthy lungs, one from a patient with fibrosis, one from a patient with fibrosis and emphysema, and one from a patient with emphysema. In all the test cases (theoretical and real patients), the user interaction was minimal—less than 10 s for the seed-point selection, and, even if in this stage the software development was not focused on speed performance, the algorithm has shown reduced running time (under 1 min per dataset—it can be improved to seconds in future developments). The cases with emphysema were used to test the limitations of our algorithm, but even in these cases, at least with these initial tests, the method remains valid, and correct results, which can be interpreted medically and corelated with other investigations, are obtained.

The results obtained for the theoretical lung volume are shown in Figure 1—unsegmented 3D image, 2D sections, and histogram—and in Figure 2—segmented 3D image, 2D sections, and histogram. The red regions visible in the 3D part of Figure 1 have high intensities, representing pathology and blood vessels; they extend beyond the lungs and are cut off from the interior of the lungs in the segmented Figure 2. Also, the region with a ball-like shape and the same intensities as the lung tissue (in Figure 1) is removed in Figure 2 (segmentation result) because it is not connected to the tracheobronchial tree.

**Figure 4:**
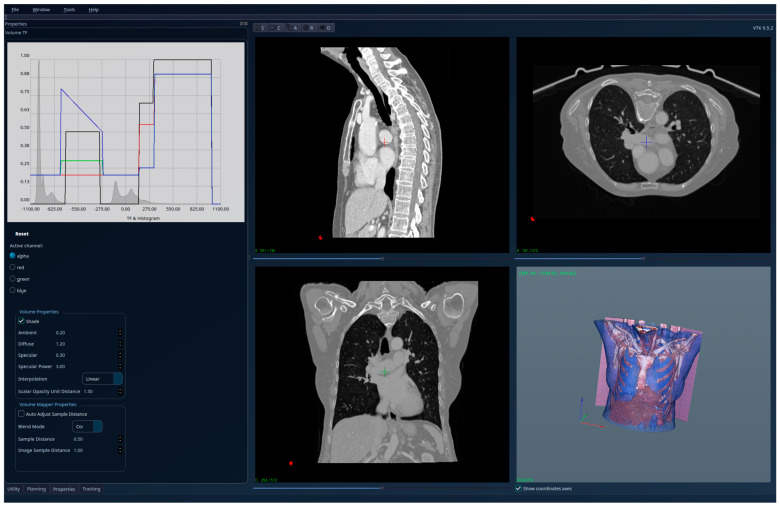
Patient 1 (healthy lungs)-unsegmented; the HU histogram displays several peaks, limiting clinically meaningful analysis.

**Figure 5:**
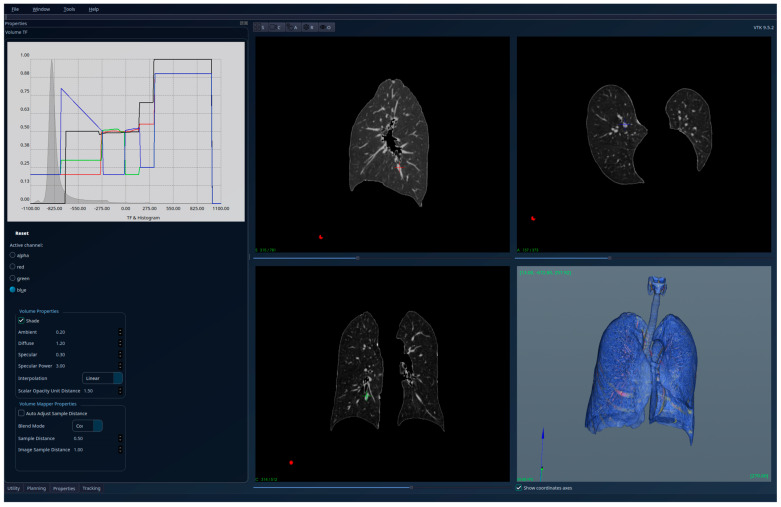
Patient 1 (healthy lungs)-segmented, with lung tissue visible; the HU histogram has only one peak in the region [−900, −800].

**Figure 6:**
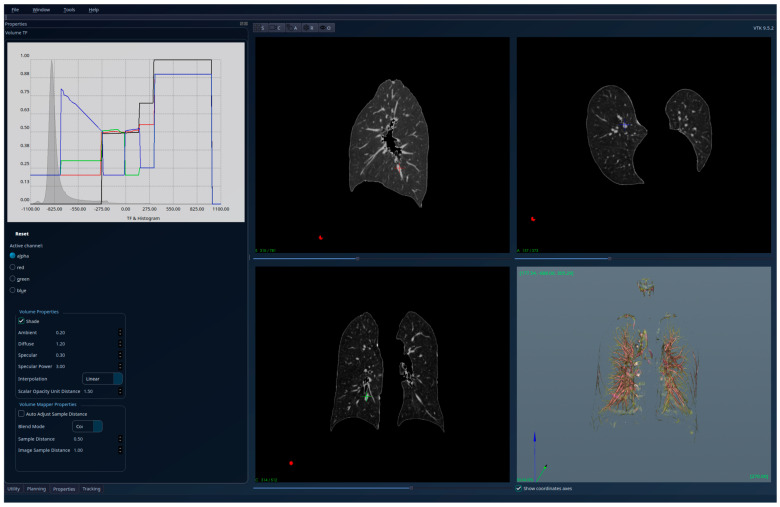
Patient 1 (healthy lungs)-segmented, with lung tissue hidden, and red regions showing the blood vessels.

The results for the patient with healthy lungs are presented in Figure 4—unsegmented 3D image, 2D sections, and histogram; Figure 5—segmented 3D image with visible lungs’ tissue, 2D sections, and histogram; and Figure 6—segmented 3D image with hidden lungs’ tissue, 2D sections, and histogram. The red regions visible in the 3D part of Figure 5 represent blood vessels inside the lungs’ volume. The histogram of the segmented image shows a peak in the region [−900, −800] and very few intensity values higher than −200.

**Figure 7:**
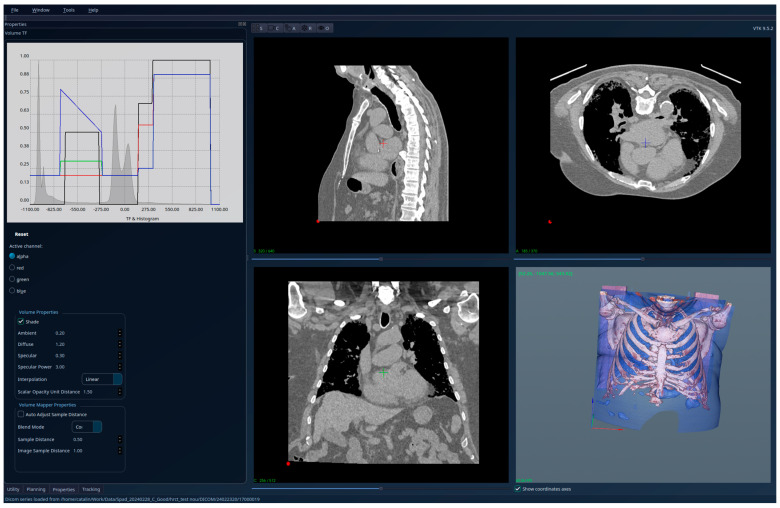
Patient 2 (fibrosis)-unsegmented; the HU histogram displays several peaks, limiting clinically meaningful analysis.

**Figure 8:**
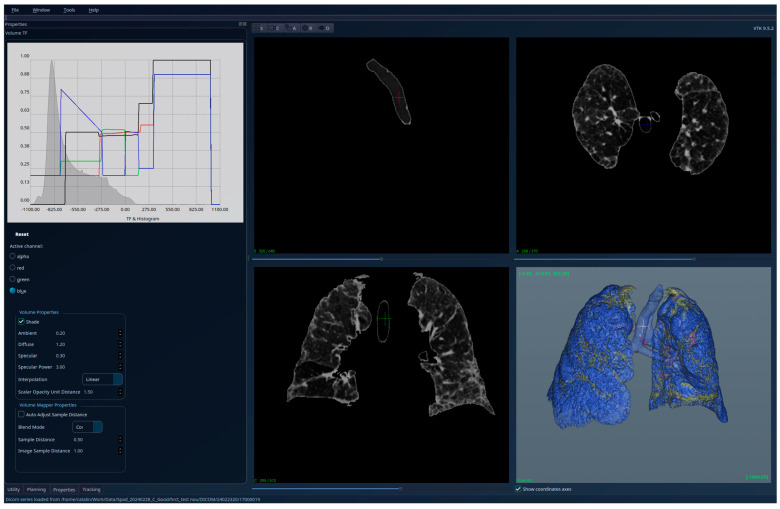
Patient 2 (fibrosis)-segmented, with lung tissue visible; the HU histogram displays one peak in the region [−900, −800] and many intensity values higher than −200.

**Figure 9:**
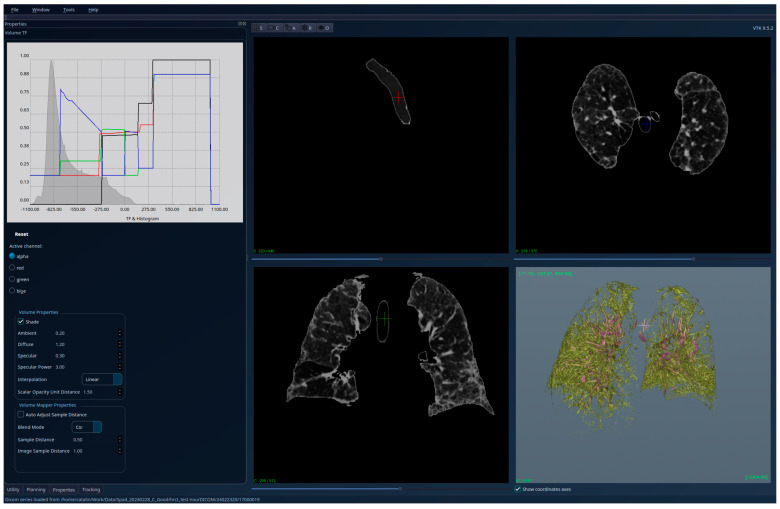
Patient 2 (fibrosis)-segmented, with lung tissue hidden; the fibrotic (yellow) regions are clearly visible in the 3D view.

The results for the patient with fibrosis are presented in Figure 7—unsegmented 3D image, 2D sections, and histogram; Figure 8—segmented 3D image with visible lung tissue, 2D sections, and histogram; and Figure 9—segmented 3D image with hidden lung tissue, 2D sections, and histogram. The red regions visible in the 3D part of Figure 8 represent blood vessels within the lung volume, while the yellow region occupying a large portion of the lung volume is fibrosis. The histogram of the segmented image shows a peak in the range [−900, −800], but also many intensity values above −200. There is a clear qualitative difference between the Patient 1 and Patient 2 segmented histograms.

Qualitative evaluation showed that the proposed approach effectively delineated lung boundaries while managing to preserve the pathology intensities inside the segmented lungs. Compared to fully automated threshold methods, it demonstrated improved qualitative robustness in the tested cases, particularly in preserving pathological intensities within the segmented lung volume, against image artifacts and variable illumination.

**Figure 10:**
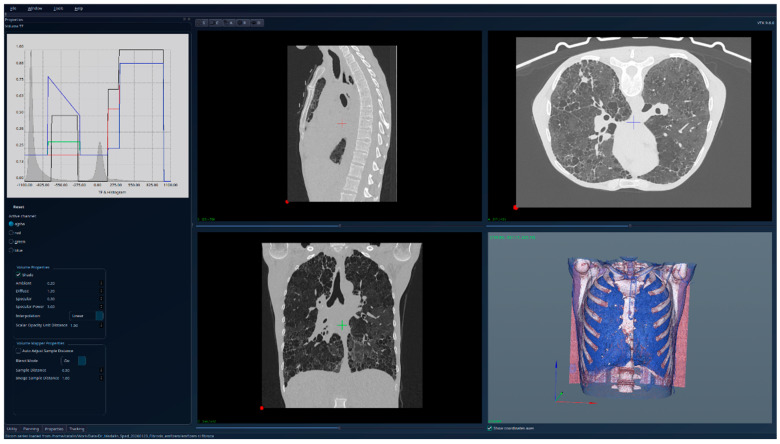
Patient 3 (fibrosis and emphysema)-unsegmented; the HU histogram displays two peaks, but the presence of image artifacts (e.g., patient support) and of tissues outside the lung volumes limits its usefulness.

**Figure 11:**
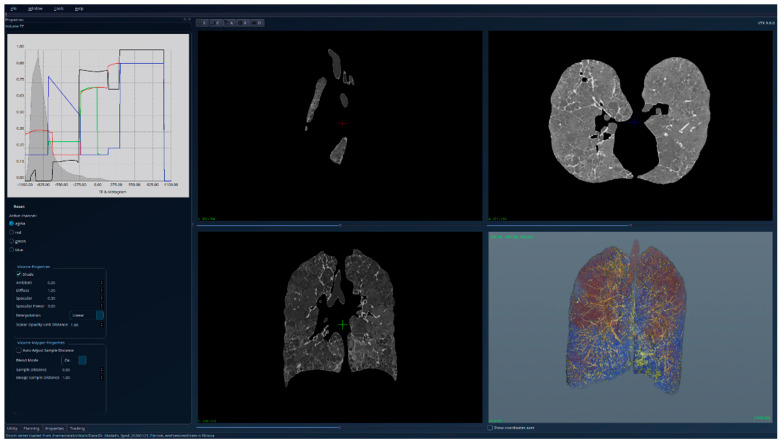
Patient 3 (fibrosis and emphysema)-segmented, with lung tissue and emphysema regions (reddish areas) visible with high transparency; the HU histogram shows one peak around the value −1000 HU, and the emphysema causing the displacement of this peak to the left compared to healthy lungs.

**Figure 12:**
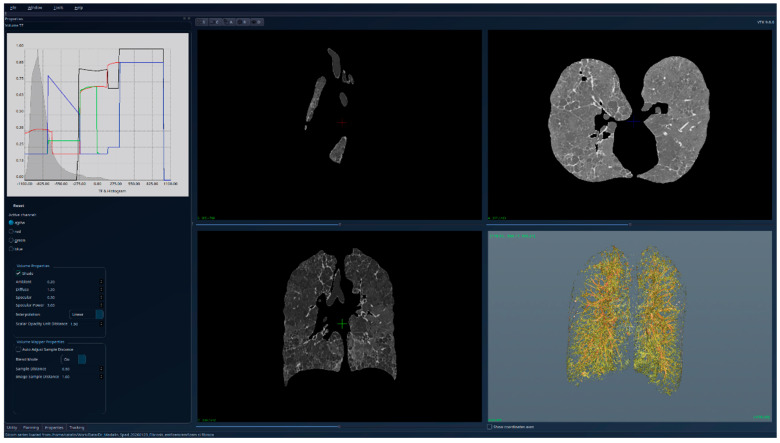
Patient 3 (fibrosis and emphysema)-segmented, with lung tissue and emphysema regions hidden, while the fibrosis is clearly visible in 3D.

Figure 10, Figure 11 and Figure 12 present the results for a patient affected by fibrosis and emphysema, a case used for testing the limitations of the algorithm. But even in this case, the algorithm produced correct results, correlated with other medical investigations and tests, and the histogram can be interpreted from the medical perspective, indicating the presence of emphysema. Also, the presence of fibrosis, as in the previous cases with only fibrosis, can be detected visually directly in the segmented 3D image, and by analyzing the right side of the HU histogram.

**Figure 13:**
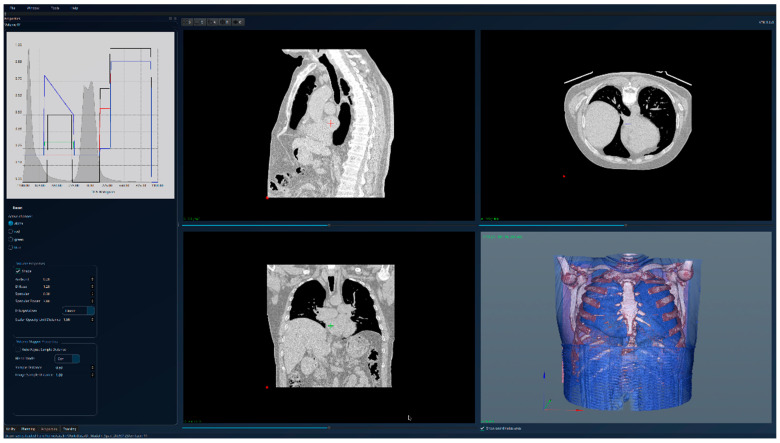
Patient 4 (emphysema)-unsegmented; the HU histogram displays two peaks, but the presence of image artifacts (e.g., patient support) and of tissues outside the lung volumes’ limits cannot be interpreted in the context of lung diseases.

**Figure 14:**
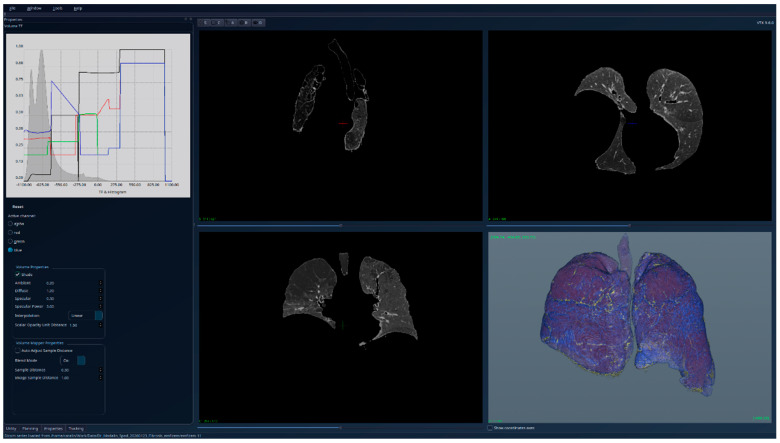
Patient 4 (emphysema)-segmented, with lung tissue and emphysema regions (reddish areas—larger than in Patient 3’s case)—visible with high transparency; the HU histogram shows one peak around the value −1000 HU, with the emphysema causing the displacement of this peak to the left compared to healthy lungs.

**Figure 15:**
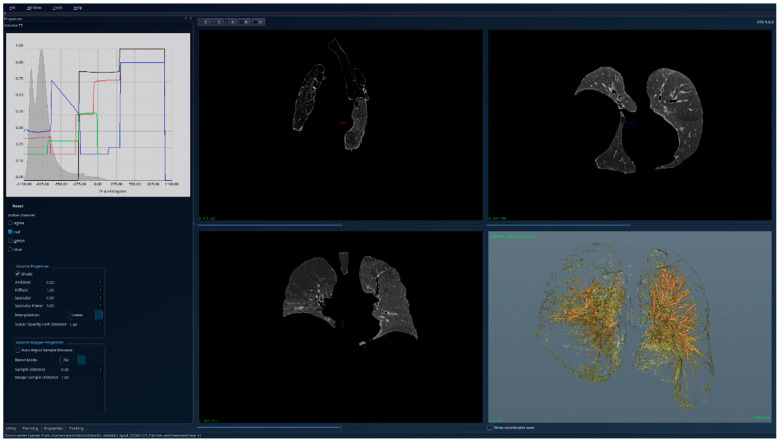
Patient 4 (emphysema)-segmented, with lung tissue and emphysema regions hidden, blood vessels are now visible in 3D, and also an area with fibrosis could be seen, but it is less extended than in Patient 3’s case.

Figure 13, Figure 14 and Figure 15 present the results for a patient affected by emphysema, another case used for testing the limitations of the algorithm. The initial conclusion is the same as for the “fibrosis and emphysema case”—our region-growing algorithm is still valid and the HU histogram of the segmented volume has medical significance, with clear signs of the emphysema.

The running times were obtained on a mobile workstation: CPU Intel 12th Gen i9-12900H, 32 GB RAM, Nvidia Geforce RTX 3070 Ti Laptop GPU.

As shown in Table 3, all real datasets were reconstructed using the Br60 convolution kernel. This choice represents a practical compromise between spatial detail and image noise. More aggressive high-frequency kernels (e.g., Br64) introduced substantially increased noise, which first degraded the stability and interpretability of the resulting intensity histograms and subsequently promoted region-growing leakage into surrounding tissues, particularly adipose regions.

### Code Snippets

The results described so far were obtained in a new module implemented for the software platform described in [22]. The implementation is based on C++/VTK/CTK, and it follows the guidelines from this pseudo-code:

*function embeddedRegionGrowing(*

*lungRange, // [min, max] value interval to consider “lung” (original: [−1500, −200])*

*initValue, // background fill value for result volume (original: −5000)*

*maxRaySteps, // 5 in original*

*maxMisses // 2 in original*

*):*

*start timer*

 

*sourceVolume = getCurrentVolume()*

*resultVolume = createEmptyVolumeLike(sourceVolume)*

 

*// Fill result volume with a default value*

*parallel_for each sliceRange in volumeDepth:*

*for each voxel (x,y,z) in sliceRange:*

*resultVolume[x,y,z] = initValue*

 

*seed = getSeedPoint()*

 

*visited = set()*

*queue = FIFO()*

*enqueue seed; mark visited*

 

*function isEmbeddedInLung(voxel):*

*misses = 0*

*for each of 6 axis directions:*

*if not rayHitsRange(voxel, direction, maxRaySteps, lungRange):*

*misses += 1*

*if misses > maxMisses: return false*

*return true*

 

*histogram = empty map*

*while queue not empty:*

*voxel = queue.pop()*

 

*if not isEmbeddedInLung(voxel):*

*continue*

 

*value = sourceVolume[voxel]*

*histogram[value] += 1*

*resultVolume[voxel] = value*

 

*for each 6-connected neighbor of voxel:*

*if neighbor not in visited:*

*mark visited*

*enqueue neighbor*

 

*replaceCurrentVolume(resultVolume)*

*log elapsed time*

 

The Dice scores between Slicer reference segmentations and our results were obtained with this Python 3.14.2 code, based on SimpleITK 2.5.3 and numpy 2.3.5:

*import SimpleITK as sitk*

*import numpy as np*

 

 

*def compute_dice(path_slicer_ref, path_segm):*

*img1 = sitk.ReadImage(path_slicer_ref)*

*img2 = sitk.ReadImage(path_segm)*

 

*if img1.GetSize() != img2.GetSize():*

*print(“Images have different sizes:“)*

*print(f”Image 1 size: {img1.GetSize()}”)*

*print(f”Image 2 size: {img2.GetSize()}”)*

*return*

 

*arr1 = sitk.GetArrayFromImage(img1) != −3000 # Slicer segmentation background*

*arr2 = sitk.GetArrayFromImage(img2) != −5000 # Our segmentation background*

 

dice = 2.0 * np.logical_and(arr1, arr2).sum() / (arr1.sum() + arr2.sum())

*print(f”Dice: {dice}”)*

## 4. Discussion

The present study demonstrated that the semi-automated region-growing segmentation approach accurately identifies lung volume while preserving all parenchymal intensities, including vessels and pathological elements such as fibrotic tissues. Compared to conventional threshold-based segmentation approaches, this is a significant advantage, since the latter do not account for smoothing, or only partially include the non-air portion of the lung parenchyma [24,25]. While preserving the entire internal intensity distribution of the segmented lung volume, the presented method enables post-segmentation histogram analysis and provides a meaningful framework for designing a future quantitative fibrosis score [26,27].

One of the best things about the suggested procedure is that it is partially automated, which is very helpful for interstitial lung disease. ILD often shows fibrosis, traction bronchiectasis, subpleural reticulation, and airway distortion, which alter attenuation values and create uneven anatomical borders. In these cases, fully automated methods often do not work, which can damage the soft tissues in the mediastinum or pleura or not properly separate the affected lung areas [8].

Region-growing methods are particularly suited for lung parenchyma segmentation because they exploit the local continuity of tissue intensity [25]. In the present implementation, the addition of an “embeddedness” filter represents an important conceptual contribution, allowing the algorithm to include voxels of any intensity as long as they are structurally contained within the lung volume. This strategy prevents exclusion of fibrosis or vascular elements while cutting protruding components that extend beyond lung boundaries [14]. Qualitative evaluation on both theoretical and real datasets confirmed that the method preserved pathological intensity ranges without artificial truncation, which is essential for downstream quantitative analysis [24].

Compared with classical threshold-based or edge-detection segmentation, the proposed technique demonstrated greater tolerance to intensity variability and image artifacts. At the same time, unlike deep learning-based segmentation frameworks, the method does not rely on large, annotated training datasets and behaves in a transparent, interpretable manner. For research and planning workflows, such interpretability is advantageous, particularly when segmentation results are subsequently used to derive quantitative imaging biomarkers or to support clinical decision-making.

There are still some methodological limitations that must be acknowledged. The current implementation was tested only on a small number of real patient datasets, primarily those exhibiting typical interstitial lung disease morphology. Although performance was strong in these cases, severe pathologies—such as large tumors, massive pleural effusions, or complex post-surgical alterations—could challenge region-growing propagation and boundary stability [12]. Region-growing methods excel in homogeneous regions but struggle with complex anatomical geometries, intensity inhomogeneities, and conditions like pleural effusions or consolidations, potentially yielding suboptimal segmentation or false negatives [12,28].

Another drawback is that segmentation still requires manual seed selection, even though the time users spend is very short. This design choice was made deliberately to maintain control in complex pathological cases. However, future improvements may include automatic seed estimation or hybrid deep learning refinement modules to further improve automation without making it harder to understand [29].

From a clinical perspective, the algorithm’s ability to preserve the entire internal intensity spectrum of lung volume is very important [27,30]. The differences observed between the histograms of healthy and fibrotic lungs suggest that histogram features could serve as potential imaging biomarkers for assessing disease burden or progression [26]. In the current implementation, the distinction between COPD-dominant and ILD-dominant phenotypes is not performed as a categorical classification step; rather, it is addressed through analysis of post-segmentation attenuation distributions. COPD-dominant lungs usually have a histogram that shifts to the left and has a lot of very low attenuation values (for example, below −950 HU), which shows emphysematous destruction. On the other hand, ILD-dominant lungs exhibit a relative increase in higher attenuation values (above −200 HU), indicating ground-glass opacities, reticulation, and fibrosis [31]. Mixed phenotypes may show features from more than one histogram. This attenuation-based characterization provides a quantitative framework that may facilitate the differentiation of disease patterns in subsequent advancements. Interstitial lung disease is primarily characterized by increased-attenuation patterns such as ground-glass opacities, reticulation, traction bronchiectasis, and fibrosis, whereas COPD is dominated by low-attenuation emphysematous destruction and air trapping [32]. The suggested embeddedness-based segmentation strategy aims to maintain voxels of any intensity, provided they are structurally integrated within the lung volume. However, these intrinsic pathophysiological disparities may result in disease-specific segmentation behavior. Subsequent studies should incorporate stratified analyses by disease category to better characterize performance across ILD, COPD, and mixed phenotypes, thereby optimizing parameter settings accordingly.

Overall, the suggested method strikes a good balance between manual and fully automated segmentation. It works well in pathological lungs, requires little computing power, and is well-suited for CAD systems and quantitative imaging applications. Future work will focus on large-scale validation, integrating adaptive thresholds and connectivity analysis, and creating quantitative scores specific to fibrosis using the preserved post-segmentation intensity distributions.

### 4.1. Limitations

This study has several important limitations that should be acknowledged. First, the number of real patient datasets used for validation is limited and does not allow broad generalization of the results. Although the qualitative behavior of the algorithm was consistent across the tested cases, larger and more diverse cohorts are required to fully characterize performance across the wide spectrum of interstitial lung disease phenotypes and severities.

Second, the present study does not include voxel-wise ground-truth annotations created by human experts. Manual delineation of lung boundaries in advanced fibrotic lungs is inherently imprecise and subject to high inter- and intra-observer variability, which complicates the establishment of a reliable reference standard. Although overlap-based metrics such as the Dice similarity coefficient were included in the current evaluation, the absence of manual expert ground-truth annotations remains a limitation. Automated reference segmentations were used to ensure reproducibility; however, consensus-based expert annotations would further strengthen future validation studies. Third, the method remains semi-automated, requiring manual placement of a single seed point inside the trachea. Although this step typically requires only a few seconds and is unlikely to introduce major variability, it still represents a degree of user dependency. Automated or hybrid seed initialization strategies may further improve usability.

Fourth, the algorithm was primarily evaluated on HRCT datasets reconstructed using a single convolution kernel. Differences in acquisition protocols, reconstruction kernels, slice thickness, and scanner vendors may influence image noise characteristics and segmentation behavior. Multicenter validation across heterogeneous imaging protocols is therefore necessary.

### 4.2. Future Work

Future research will focus on large-scale quantitative validation of the proposed segmentation method using multicenter datasets and heterogeneous acquisition protocols. Comparative benchmarking against established conventional segmentation tools and deep learning-based methods will be performed using overlap-based metrics and surface distance measures.

In addition, automated or hybrid seed-point initialization strategies will be investigated to further reduce user interaction. Integration of the embeddedness-based criterion into learning-based refinement frameworks may combine interpretability with improved boundary accuracy. One other future step on the software development side will be to improve the running time, taking advantage of parallel computing (CPU multithreading, Cuda). In the current development, we used only a single CPU thread, with already much better time performance (less than one minute per dataset) than AI-based methods, so only a few seconds, or even less than a second, of running time is a realistic target.

Finally, histogram-derived features extracted from the segmented lung volume will be explored as potential quantitative imaging biomarkers for fibrosis burden and longitudinal disease progression, and they are correlated with pulmonary function tests and clinical outcomes.

## 5. Conclusions

The semi-automated 3D region-growing approach to lung segmentation presented in this study offers a useful trade-off between manual tracing and fully automated techniques, with the advantages of preserving the full range of intensity information inside the lung volume of interest (enabling anatomical delineation and quantification) and requiring a low level of operator interaction. The method appeared to perform well in patients with interstitial lung disease, where the complex, heterogeneous lung parenchymal attenuation and the disturbed lung morphology might affect the accuracy of traditional threshold-based or fully automated segmentation methods. Due to its simplicity, intuitiveness, and low computational demand, the method is directly applicable in research studies, quantitative imaging applications, and computer-aided diagnosis.

Additionally, the segmented volume can be used as a robust preprocessing step for subsequent tasks, such as 3D visualization, airway and vessel analysis, nodule or lesion detection, volume measurement, or disease severity assessment. Moreover, including vessels and disease within the segmented volume enables post-segmentation histogram analysis, which may be useful for developing and validating fibrosis-related quantitative imaging biomarkers and follow-up tools in ILD.

As a future direction, we will seek to further validate our method on larger and more varied multicenter datasets, including cases with severe and/or atypical disease. We will also investigate the inclusion of adaptive similarity criteria, 3D connectivity criteria, and automated seed-point placement to reduce the method’s sensitivity and increase its robustness against operator variability. We will explore using guidance from deep features or modules to refine the segmented boundary via learning, thereby increasing the method’s accuracy while maintaining its explainability. Finally, we will focus on deriving quantitative (fibrosis) scores from the histogram of post-segmentation preserved intensity information and on evaluating their value as imaging biomarkers in a clinical and research context.

## Figures and Tables

**Table 1:** Dice score computed between Slicer reference segmentation and our algorithm.

Dataset	Slicer Segmentation Method	Dice Score
Theoretical lungs model	Lung CT Segmenter—TotalSegmentator lung extended	
Patient 1 (healthy lungs)	Lung CT Segmenter—TotalSegmentator lung extended	0.9699
Patient 2 (fibrosis)	Lung CT Segmenter—TotalSegmentator lung extended	0.9579
Patient 3 (fibrosis, emphysema)	Lung CT Segmenter—TotalSegmentator lung extended	0.9795
Patient 4 (emphysema)	Lung CT Segmenter—TotalSegmentator lung extended	0.9738

**Table 2:** Datasets used for tests—voxel count and size, and algorithm running times.

Dataset	Voxels (Count)	Voxels (Size—mm)	Segmentation Duration (Milliseconds)
Theoretical lungs model	512 × 512 × 512	0.5 × 0.5 × 0.5	5227
Patient 1 (healthy lungs)	781 × 512 × 373	0.5 × 0.5 × 1	58,696
Patient 2 (fibrosis)	640 × 512 × 370	0.6 × 0.6 × 1	38,113
Patient 3 (fibrosis, emphysema)	709 × 512 × 433	0.47 × 0.47 × 0.8	115,302
Patient 4 (emphysema)	621 × 512 × 498	0.6 × 0.6 × 0.8	54,926

**Table 3:** Datasets DICOM information—convolution kernel, slice thickness, manufacturer, and model name.

Dataset	Kernel	SliceThickness	Manufacturer	Model
Theoretical lungs model				
Patient 1 (healthy lungs)	Br60	1.5	Siemens Healthineers	SOMATOM go.Top
Patient 2 (fibrosis)	Br60	1	Siemens Healthineers	SOMATOM go.Top
Patient 3 (fibrosis, emphysema)	Br60	1	Siemens Healthineers	SOMATOM go.Top
Patient 4 (emphysema)	Br60	1	Siemens Healthineers	SOMATOM go.Top

## Data Availability

Due to privacy and ethical considerations, the datasets generated and/or analyzed during the current study are not publicly available but may be provided upon reasonable request.
